# Patient with McCune albright syndrome: Case report and 10 Years of follow-up imaging examination

**DOI:** 10.4317/jced.60161

**Published:** 2023-03-01

**Authors:** Thaygla-Cristhina-Araujo Gandra, Isabella-Caroline-Fonseca Tavares, Ana-Luiza-Farnese-Morais Carlos, Lizandra-Gonzaga Rodrigues, Izabella-Lucas-de Abreu Lima, Flávio-Ricardo Manzi

**Affiliations:** 1 Graduating, Department of Dentistry/Oral Radiology, School of Dentistry, Pontifical Catholic University of Minas Gerais, Belo Horizonte, Brazil; 2 Masters Student, Department of Dentistry/Oral Radiology, School of Dentistry, Pontifical Catholic University of Minas Gerais, Belo Horizonte, Brazil; 3 Professor Doctor, Department of Dentistry/Oral Radiology, School of Dentistry, Pontifical Catholic University of Minas Gerais, Belo Horizonte, Brazil

## Abstract

The McCune Albright syndrome (MAS) is a rare, multi-system disease composed of the triad of polyostotic fibrous dysplasia of bone (PFDB), café-au-lait skin hyperpigmentation, and endocrine disorders. The diagnosis involves clinical, biochemical and imaging findings, with dentistry playing an important role in MAS, since many patients present DFPO in the craniofacial bones, including the maxilla and mandible, and in view of their dental needs, the correct management of these patients is not only an essential but important area to be investigated. This report presents a case of a patient with McCune Albright Syndrome, the behavior of the disease over a period of 10 years and how imaging exams such as scintigraphy and tomography were important for planning the dental treatment of this patient, since they are fundamental allies for identification and evaluation of the progression and/or stability of the disease.

** Key words:**Craniofacial fibrous dysplasia, cone-beam computed tomography, scintigraphy, imaging diagnosis.

## Introduction

McCune Albright Syndrome (MAS) is a multisystem disease, defined by the triad: polyostotic fibrous dysplasia of bone (PFDB), café-au-lait skin hyperpigmentation and various endocrine disorders (1). This condition is considered rare, with estimated prevalence between 1/100,000 and 1/1,000,000 (2). The etiology of MAS is defined by somatic activating mutations in the gene encoding an alpha subunit of the binding protein (GNAS) to the guanine nucleotide stimulator (GαS) and lead to activation of the constitutive receptor and dysregulated production of cyclic adenosine monophosphate (cAMP), an important regulator of cellular metabolism (2,3). The result of this genetic mutation is a mosaicism with a broad clinical spectrum, and the specific manifestations depend on the tissues that carry the pathogenic variant (4,5).

Activation of the G protein-coupled receptor triggers the production of various hormones and prolonged signaling of GαS in the endocrine organs. This can trigger precocious puberty (PP), hyperthyroidism, excessive growth hormone (GH) and other endocrinopathies (5). Furthermore, MAS is associated with skin blemishes, characterized by café-au-lait hyperpigmentation with irregular borders. This occurs as a result of the increased expression of the tyrosinase gene and the production of melanin by the melanocytes affected (5). Individuals with MAS may also manifest altered signaling in bone tissue that leads to inadequate proliferation of immature osteoblastic progenitor cells, producing dysplastic lesions in the bone. This change characterizes PFDB, in which normal bone is replaced by fibro-osseous tissue, leading to weakening of the skeleton and predisposing it to fractures, deformities, pain and functional impairment (5,6).

The diagnosis of MAS is based on clinical, biochemical and radiographic findings (7). The recommendation is that all individuals with suspected MAS should be submitted to analysis of their thyroid and pituitary activities, insulin growth factor, and in children the speed of growth should be monitored, since the endocrine manifestations of MAS can cause changes in growth (7). In children aged 5 years and older, bone scintigraphy exam with application of the radiopharmaceutical technetium (99m Tc-methylene diphosphonate) may also be useful for identifying areas of PFDB. This can later be evaluated in radiographic exams, in which the dysplastic areas will be shown to have a homogeneous ground glass’ appearance (7). Activity of the disease tends to decrease during the adult phase, thus, from a radiographic aspect, PFDB assumes a more complex, sclerotic and heterogeneous appearance (7).

Dentistry plays an important role in MAS since approximately 90% of patients with PFDB have lesions in the craniofacial bones, including the maxilla and mandible (7). Dysplastic bone lesions are capable of growing rapidly, causing facial disFigurement due to bone expansion and displacement of adjacent structures, such as the orbit and teeth (7). They may also be associated with dental disorders such as enamel hypoplasia, dentin dysplasia, taurodontism, odontoma, malocclusion and high rate of caries (7). The dental management of patients with MAS becomes complex due to the presence of medical comorbidities that include skeletal disease, endocrine disorders, the use of various medications and general weakness, therefore, the dental aspects are often neglected (7). Considering the importance of dental management of these patients, the aim of this study was to report the case of a patient diagnosed with MAS and behavior of the disease over the course of a period of 10 years of follow-up. 

## Case Report

**Figure 1:**
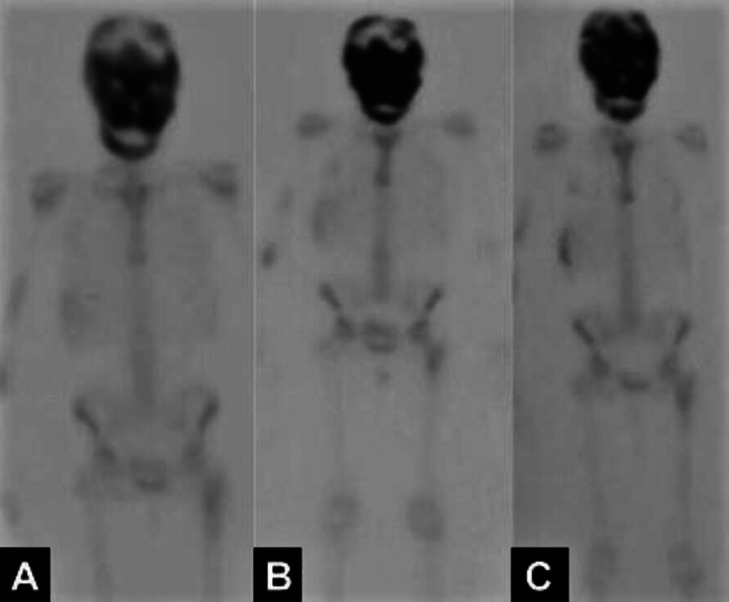
A. Scintigraphy performed at age 16, showing abnormal hyperuptake in certain areas of the patient’s body, especially the skull. B. Examination performed at age 22 years showing only an increase in the intensity of radiopharmaceutical concentration in the topography of the skull, with no new areas of anomalous hyperuptake. C. Examination performed at age 26 without considerable changes in disease progression, demonstrating stability.

**Figure 2:**
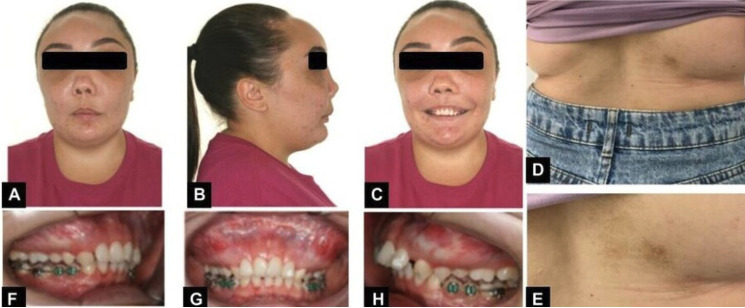
A,B,C) Front view, profile and smile of the patient. D,E) Café au Leite stain on the right side of the patient’s back. F,G,H) Increase in Maxillary Volume Noted on Intraoral Examination.

**Figure 3:**
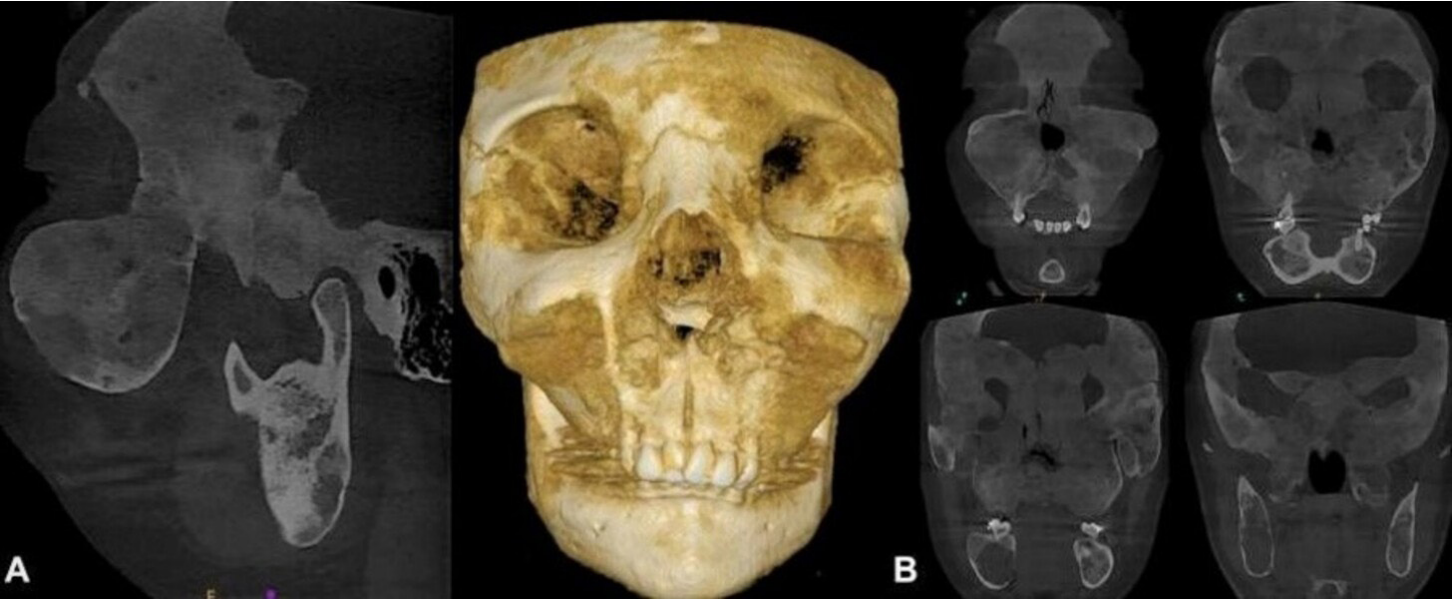
A. Bone lesions caused by Polyostotic Fibrous Dysplasia of Bone. B. Maxillary involvement with total aplasia of the maxillary sinuses.

The patient, a 26-year-old Caucasian woman was referred by the oral and maxillofacial surgeon for orthodontic treatment in order to prepare for orthognathic surgery, with the aim of correcting the changes in growth of the maxillae. During the anamnesis, the patient reported that at about two years of age, her mother took her to the doctor, since she frequently suffered from a blocked nose, however, the pediatrician attributed the problem to allergic episodes. Only at the age of five, when extrusion of the left eyeball was noted, were CT scans requested, resulting in the diagnosis of MAS. After the examination was performed, the cause of the complaint of a blocked nose was confirmed to be airway obstruction due to the disease. Moreover, the patient reported that she had menarche at 11 years of age. During adolescence and early adulthood, scintigraphy examinations were requested to assess progression of the condition. The first scintigraphy exam was performed at age 16. This showed abnormal hyperuptake in the areas of the skull, mandible, distal portion of the right humerus, proximal portion of the right ulna, posterior portions of the right and left costal arches, and proximal portion of the left femur (Fig. 1A). At 22 years of age, she repeated the examination, in which no new areas of anomalous hyperuptake were observed. There was only a slight increase in the intensity of the radiopharmaceutical concentration in the topography of the skull (Fig. 1B). At 26 years of age, the examination was performed again and no changes were found when compared with the examination performed at age 22, demonstrating stability of the process (Fig. 1C). When the patient was still at the stage of adolescence, maxillofacial surgeon performed some procedures to correct the abnormalities the patient presented, as follows: 1 craniofacial surgery for decompression of the optic nerve and 4 nose surgeries for unobstructing the airways, the latter surgery was performed at 19 years of age. It is worth emphasizing that the patient has been followed up by an endocrinologist, otorhinolaryngologist, neurologist and orthopedist since she was 5 years old. In the extraoral clinical examination, it was noted that the patient had facial asymmetry (Fig. 2A-C) and an exacerbated bone development in the maxilla when compared with the mandible. In addition, she reported some pain in the region of the maxillary arch. The patient also reported that she had a single café au lait stain in the region of her back (Fig. 2D,E) . On intraoral examination, a more evident increase in volume was observed in the maxillary arch, affecting the maxillary bone (Fig. 2F,G). In view of the clinical findings, patient’s reports, and her medical history, a CT scan was requested to assess the patient’s bone pattern. Cone-beam computed tomography was performed with the CS 9600-3D device (CarestreamDental, Atlanta, USA), parameters of 120 kV, 8 mA, 150 um Voxel and 16.0 cm x 17.0 cm FOV. The digital images were stored in the KDIS system - Kodak Dental Imaging Software (Carestream Health Inc., New York, USA) in the original format (DICOM). In the tomographic reconstructions, an expansive lesion was observed, going in all directions with varying degrees of bone density (numerous bone trabeculae irregularly arranged with hypodense areas - ground glass appearance), affecting the following bones: Frontal, Ethmoid, Temporal, Zygomatic, Sphenoid, Lower Nasal, Maxillary and Mandibular Bone (Fig. 3A). With specific regard to the maxilla, the lesion affected the frontal, zygomatic, palatine and alveolar processes, maxillary tuberosity, in addition to the body regions of the maxilla, with a total aplasia of both maxillary sinuses and consequent total obliteration of the ostiomeatal units (Fig. 3B). In the mandible, the lesion involved the entire region of the symphysis, mandibular body on both sides, retromolar trigone, angle and ascending rami, with displacement of the mandibular canals bilaterally and of the mental foramina to the superior region. There was also a large multilocular hypodense area in the region of the body of the mandible, angle and inferior portion of the ascending ramus of the mandible on the right side, suggestive of an area of fibrous connective tissue. Relative to the airways, the exam showed a constriction in the patient’s pharyngeal region, adjacent to the posterior-inferior portion of the soft palate, suggesting a possible retrusion of soft tissue. In view of the foregoing description, with the results of the up-to-date tomography and scintigraphy exams confirming the stability of disease progression, by taking the necessary precautions and being aware of the limitations of the treatment imposed by bone, it will be possible to continue with the orthodontic treatment.

## Discussion

The diagnosis of MAS was correlated with the clinical findings, by follow-up of the skin lesions, tomographic diagnosis of bone lesions and confirmation of endocrine disorders (6). Sometimes, the café au lait stains are the first noticeable changes. of the syndrome that appear at birth or shortly after it (6,8). However, in the present case, the respiratory and ocular changes were the features that prompted the child’s mother to seek medical help. Clinically significant bone lesions are usually apparent by 5 years of age and tend to expand during childhood and adolescence (9). Moreover, precocious puberty is common in these patients and should be treated early by an endocrinologist, as endocrine dysfunctions that exacerbate skeletal injuries may occur (6,8,9).

In addition, it could be pointed out that craniofacial PFDB was capable of causing severe nasal congestion, due to involvement of the turbinates, nasal obstruction and obliteration of the maxillary sinuses (9). Thus, airway obstruction observed in the present case corroborated the findings of reports published in the literature. Therefore, it is important for these patients to be evaluated routinely and in the long-term for the purpose of monitoring the progression or stability of the lesion, with computed tomography being an exam to be performed periodically (6,9). In the present case, the tomography required was relevant for assessment of the patient’s dysplastic bone pattern, thereby allowing important information to be obtained for determining the feasibility of performing the orthodontic movement.

Relative to scintigraphy, an exam that can help to establish the presence and extent of bone involvement in patients suspected of having or diagnosed with the syndrome. Moreover, it is a more sensitive tool for early detection and an important ally in long-term follow-up of the disease (10-12). As shown by means of bone scintigraphy analysis, the areas most commonly affected by PFDB are the base of the skull, jaw, facial bones, femur and legs, as in the case of the patient in question (10).

Furthermore, in patients diagnosed with MAS, craniofacial involvement is frequently found, therefore, bone pain may be present and can compromise not only the quality of life of these individuals but lead to varying degrees of facial asymmetry (9). Thus, due to dysplasia that can affect both maxillary and mandibular bone, these patients have a predisposition to malocclusion that sometimes requires orthodontic treatment (9). In the case reported, the patient needed orthodontic treatment and complained of pain in the jaw bone. Provided that the disease is followed-up, these patients can undergo routine dental and orthodontic treatment without worsening the prognosis of their craniofacial injuries (4).

When orthodontic therapy is indicated, the time to begin with treatment must be discussed. It is often advisable to wait until the patient reaches skeletal maturity (7). It is worth noting that the reports in the literature relative to the development of orthodontic treatment in these patients are still unclear. One theory states that orthodontic movement in these patients tends to be fast, however, depending on the patient, it can take even longer than in the normal population and relapse is more common (7). Therefore, no consensus has yet been reached on this aspect. Orthognathic surgery can be performed in conjunction with orthodontics and can help not only to restore sTable and functional occlusion, but also contribute to facial esthetics. Please note that it should be avoided in patients during the active phase of growth (4,9). In the present case, the patient has an indication for orthognathic surgery. However, a waiting time is being respected so that it will be performed only in the adult phase, outside of the period of greatest growth. The finding of stability of the disease was confirmed by the imaging tests required and comparison with the previous exams, thus enabling implementation of the treatment plan.

With regard to management of MAS, this can be affirmed to be complex and challenging. It must be individualized, and requires a multidisciplinary team approach (1,8). This team can be composed of dentists, endocrinologists, orthopedists, physical therapists, neurologists, dermatologists, maxillofacial surgeons and otorhinolaryngologists (1). The work of these professionals is often palliative, with the aim of improving function, reducing pain, and minimizing morbidity related to deformities (6,8). In the present case, the patient is being provided with multidisciplinary follow-up. However, although a total of 4 surgeries can be reported for correcting these abnormalities, it should be pointed out that there are no proven medical therapies for the treatment of PFDB or that can change the course of the disease (6,8,9). Finally, it can be shown that it is necessary for detailed information about MAS to be passed on to the patient and family, as it is a rare syndrome. This will enable professionals to support them and help them to develop their skills according to each case, their individual limitations and characteristics (4).

In conclusion, it is important for the dentist to be aware of the characteristic signs of MAS for possible detection of the disease at an early stage. From the diagnosis, follow-up of the patient until his/her skeletal maturity is reached, is of paramount importance, since the DFDB tends to stabilize when the individual reaches adulthood. From this aspect, imaging exams are fundamental allies for the identification, evaluation of the progression and/or stability of the disease, as in the case reported, in which the analysis of the patient’s previous and up-to-date exams proved to be crucial in the decisions about dental interventions. It is worth pointing out that there are still many questions regarding the correct and ideal management of the syndrome in relation to dental treatment, however, one fact is certain, for McCune Albright Syndrome the professional must know how to work with a team to favor development of the most suiTable treatment strategies that may possibly be available.
